# Dosimetric comparison using different multileaf collimeters in intensity-modulated radiotherapy for upper thoracic esophageal cancer

**DOI:** 10.1186/1748-717X-5-65

**Published:** 2010-07-15

**Authors:** Youling Gong, Shichao Wang, Lin Zhou, Yongmei Liu, Yong Xu, You Lu, Sen Bai, Yuchuan Fu, Qingfeng Xu, Qingfeng Jiang

**Affiliations:** 1 Department of Thoracic Oncology, Cancer Center, West China Hospital, Sichuan University, Chengdu 610041, Sichuan Province, China; 2 Radiation and Physics Center, Cancer Center, West China Hospital, Sichuan University, Chengdu 610041, Sichuan Province, China

## Abstract

**Purpose:**

To study the impacts of multileaf collimators (MLC) width [standard MLC width of 10 mm (sMLC) and micro-MLC width of 4 mm (mMLC)] in the intensity-modulated radiotherapy (IMRT) planning for the upper thoracic esophageal cancer (UTEC).

**Methods and materials:**

10 patients with UTEC were retrospectively planned with the sMLC and the mMLC. The monitor unites (MUs) and dose volume histogram-based parameters [conformity index (CI) and homogeneous index (HI)] were compared between the IMRT plans with sMLC and with mMLC.

**Results:**

The IMRT plans with the mMLC were more efficient (average MUs: 703.1 ± 68.3) than plans with the sMLC (average MUs: 833.4 ± 73.8) (*p *< 0.05). Also, compared to plans with the sMLC, the plans with the mMLC showed advantages in dose coverage of the planning gross tumor volume (Pgtv) (CI 0.706 ± 0.056/HI 1.093 ± 0.021) and the planning target volume (PTV) (CI 0.707 ± 0.029/HI 1.315 ± 0.013) (*p *< 0.05). In addition, the significant dose sparing in the D_5 _(3260.3 ± 374.0 *vs *3404.5 ± 374.4)/gEUD (1815.1 ± 281.7 *vs *1849.2 ± 297.6) of the spinal cord, the V_10 _(33.2 ± 6.5 *vs *34.0 ± 6.7), V_20 _(16.0 ± 4.6 *vs *16.6 ± 4.7), MLD (866.2 ± 174.1 *vs *887.9 ± 172.1) and gEUD (938.6 ± 175.2 *vs *956.8 ± 171.0) of the lungs were observed in the plans with the mMLC, respectively (*p *< 0.05).

**Conclusions:**

Comparing to the sMLC, the mMLC not only demonstrated higher efficiencies and more optimal target coverage, but also considerably improved the dose sparing of OARs in the IMRT planning for UTEC.

## Introduction

Upper thoracic esophageal carcinoma (UTEC) occurs rarely and accounts for only 5%-10% of all esophageal carcinomas in PR.China [1]. Surgery operation has not been an appropriate treatment for those with locally advanced tumors because it was difficult to achieve a clear margin. Therefore, for diseases located in the upper thoracic region, including cervical region, radiotherapy is an efficient treatment selection. To achieve a higher tumor local control, the radiation doses of 60-70 Gy to primary tumors and approximately 50-55 Gy to electively irradiated lymph nodal regions are necessary.

Due to the significant anatomical variations inside the human bodies, it has always been a challenge to deal with the target conformity and organs at risk (OARs) sparing in radiotherapy for some special kinds of tumors, including UTEC. In the last two decades, IMRT has gained wide acceptance as a state-of-art technique to treat those special tumors. It can successfully add the modulation of beam intensity to beam shaping along the radiation penetration direction and improves the dose conformity of the targets and also reduce the unnecessary radiation to the OARs [2-4].

During IMRT dose delivery, if the anatomy location between targets and OARs is very close and complicated, the MLC leaf width may have an effect on the dose distribution of the targets and the protection for OARs. Recently, several studies investigated the impacts of the MLC leaf width on the treatment planning for several kinds of tumors, and the results are controversial [5-11]. Further evaluations for the potential advantage of small leaf width on IMRT planning for UTEC are necessary.

Here, for the first time, we conducted an original study of comparing impacts of MLC leaf width in IMRT planning for UTEC. All IMRT plans were generated according to two Elekta commercial MLC devices and all of operations and software applied were performed in our therapy planning system (TPS, Philips Pinnacle^3^, Version 8.0 m, Milpitas, CA).

## Methods and Materials

### Collimators

The sMLC is the MLC device equipped in the Elekta Precise Treatment System (Elekta Oncology System, Sweden). The leaf width of this MLC is 10 mm at isocenter. It has 40 leaf pairs, upper jaws and backup jaws, covering a full 40 × 40-cm field. The total leaf travel distance is 32.5 cm. There is a minimum leaf gap across banks.

The mMLC is another commercial Elekta system installed newly in our center, the Elekta Beam Modulator™(Elekta Oncology Systems, Crawley, UK) [12]. It consists of 40 opposed pairs of leaves. Each individual leaf is capable of interdigitation and projects a width of 4 mm at the isocenter. The maximum allowable field size is 16 cm across the leaf bank and 22 cm in the direction of leaf travel.

### Patient Data

This study was conducted between March 2008 and October 2009. In total 10 patients with pathologically confirmed UTEC were retrospectively evaluated in present study. The median age of the 10 patients was 48 years old (range, 39-61 years). One woman and nine men were included. All patients were staged according to the 1997 UICC/AJCC staging system [13]. The basic and clinical characteristics of the 10 patients were summarized in Table 1. Permission to conduct the study was granted by the Research Ethics Board of the University Health Network. 6 patients received treatment with the sMLC system and 4 patients received treatment with the mMLC system.

**Table 1:** Basic and clinical characteristics of the study population (n = 10).

**Age (years)**	
Median	48
Range	39-61
**Gender**	
Male	9
Female	1
**Stage**	
T3N0M0	3
T3N1M0	4
T4N0M0	2
T4N1M0	1
**Length of Pgtv (cm)**	
Median	9.4
Range	8.3-11.5
**Volume of Pgtv (cm^3^)**	
Median	94.1
Range	62.9-121.8
**Length of PTV (cm)**	
Median	16.2
Range	15.2-17.5
**Volume of PTV (cm^3^)**	
Median	278.5
Range	228.4-327.9
**Volume of total lung (cm^3^)**	
Median	3532.6
Range	2774.2-4888.1

Pgtv: planning gross target volume; PTV: planning target volume.

### Targets Delineation and Dose Prescription

All of patients in this study underwent a dedicated helical computed tomography (CT, Siemens, Somatom Plus^4^) with 3 mm slice thickness in the supine position throughout the entire neck and thorax. The entire lungs were scanned for further plan evaluation. All patients were CT scanned during normal breathing. Six of the 10 patients were immobilized with head and neck/upper thoracic thermoplastic masks, and the rest with vacuum-locked cradles. All of the CT images of patients acquired were transferred to and registered in the TPS with a standard of couch removal and laser center localization.

All of the targets and normal tissues definitions in this study were in accordance with the RTOG 50 and 62 reports [14,15]. The gross tumor volume (GTV) included all known gross diseases (primary tumor plus grossly enlarged lymph nodes) as determined by the imaging, clinical, and endoscopic findings. The clinical target volume (CTV) included correlated lymphatic drainage regions and extended to cricothyroid membranes. It was approximately defined as the GTV plus a 3- to 4-cm margin superior to the highest extension of the tumor and a 4-cm margin inferior to the lowest extension of the tumor with a 2-cm radial margin. Uninvolved bony structures and lung tissues were kept outside the CTV. The Pgtv and PTV were defined as the GTV and CTV plus a 0.3 cm margin in all direction, respectively. The spinal cord and lungs were contoured as OARs.

The planned treatment for each patient consisted of 64.5 Gy to be delivered to Pgtv in 30 fractions, 54 Gy to PTV in 30 fractions; and the prescription dose covered at least 95% of the volume of Pgtv and PTV, respectively. The maximum tolerance doses to the critical normal structures were as follows: spinal cord 45 Gy) and lungsV_20 _less than 30% and V_30 _less than 15%.

### Treatment Planning and Optimizing

In the progress of treatment planning setup and optimizing, all of the inverse IMRT plans were generated and evaluated using TPS mentioned previously. The plans were performed on the basis of 7 coplanar beams arrangement, whose angels were 204°, 256°, 308°, 0°, 52°, 104° and 156°, respectively. As mentioned previously, the TPS of Philips Pinnacle^3 ^version 8.0 m used Direct Machine Parameter Optimization method (DMPO, Raysearch™ laboratories, Stockholm, Sweden) in IMRT plans optimizing proceeding. The IMRT plan optimizing based on two MLC devices generated with the identical dose constrains and optimization parameters. For all ten patients, the parameters were same too. The max iterations of the plan optimizing were 40, and the maximum numbers of all segments in one plan were restricted within 100. There is no limitation in the minimum MUs per segment.

### Evaluation of the DVH-based Parameters

In this study, the CI was calculated for Pgtv and PTV among plans respectively, as the equation used by Zhu *et al. *[5]: CI = Pgtv_ref_/Pgtv × Pgtv_ref_/V_ref_. The Pgtv_ref _is the overlap volume between the Pgtv and volume of prescription isodose surface. The V_ref _is tissue volume that is enclosed by the prescription isodose surface also outside of Pgtv. The prescription isodose was 95% isodose to Pgtv. The same method was applied in analysis of PTV. The higher CI is, the more conformal the plan is.

The HI for targets was defined as "HI = D_5_/D_95_", where D_5 _and D_95 _are the dose received by the 5% and 95% volumes of Pgtv and PTV. The more D_5 _and D_95 _approach each other, the steeper the target's curve in DVHs. The optimal value of the HI is 1.

The generalized equivalent uniform dose (gEUD) of the OARs was calculated by the TPS automatically.

### Statistical Analysis

The statistical analysis was performed using the SPSS software (version 13.0, Chicago, USA). All data were analyzed applying "mean ± SD". Using Student's *t*-test, a value of *p *< 0.05 (two-tailed) was considered statistically significance.

## Results

**Figure 1:**
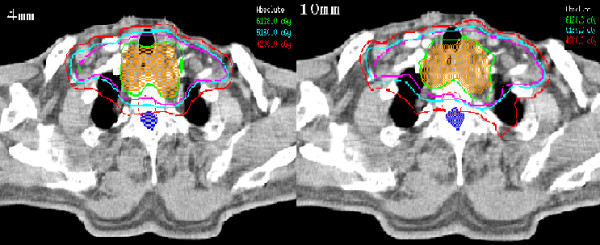
**The transverse sections of the representative IMRT plans of one patient with the irradiation isodose curves (10 and 4 mm: MLC leaf widths of 10 and 4 mm, respectively)**.

**Figure 2:**
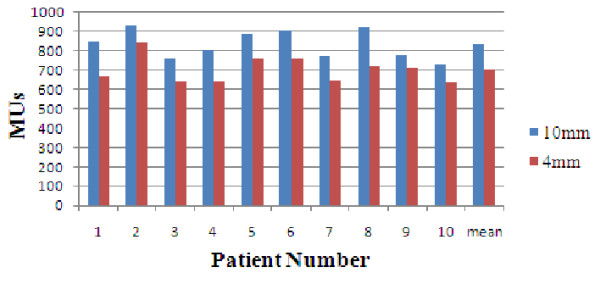
**Case-by-case comparison of the delivering MUs between the IMRT plans with the mMLC and the sMLC (10 and 4 mm: MLC leaf widths of 10 and 4 mm, respectively)**.

In total, 20 plans based on the different MLC devices were generated after the protocol and analyzed. The representative IMRT plans of one patient with irradiation isodose curves were shown in Fig. 1 and which revealed isodose curves were similar with each other. Fig. 2 showed the case-by-case comparison of the delivering MUs between these paired IMRT plans, indicating that the average MUs with the mMLC (703.1 ± 68.3) was more lower than those with the sMLC (833.4 ± 73.8) (*p *< 0.001).

### Dose Coverage of the Targets

The evaluation of the DVH-based parameters of the targets was shown in table 2. The maximum, minimum and average dose of Pgtv and the average dose of PTV were similar between these two IMRT plans, respectively, with no statistical significance (*p *> 0.05).

**Table 2:** Comparisons of the DVH-based parameters for targets in the present study (n = 10).

Targets	MLC leaf width		
	
	10 mm	4 mm	*p *value
Pgtv			
Dmax	7056.1 ± 127.2	6996.3 ± 88.9	0.24
Dmin	6035.5 ± 113.7	6132.2 ± 107.4	0.06
Dmean	6495.2 ± 39.0	6491.1 ± 30.4	0.44
PTV			
Dmean	5478.5 ± 78.4	5489.2 ± 64.3	0.74

DVH: dose volume histogram; MLC: multileaf collimator; Pgtv: planning gross target volume; PTV: planning target volume; Dmax, Dmin and Dmean: the maximum, minimum and average irradiation dose of the targets received.

The comparisons of the dose conformity for the targets in present study were summarized in Table 3. The average CI for Pgtv (0.706 ± 0.056) and PTV (0.707 ± 0.029) in IMRT plans with mMLC, were significantly better than those in plans with sMLC (for Pgtv 0.677 ± 0.086 and for PTV 0.699 ± 0.029), respectively (*p *< 0.05). Also, the average HI for Pgtv (1.093 ± 0.021) and PTV (1.315 ± 0.013) in IMRT plans with the mMLC were better than those in plans with the sMLC (for Pgtv 1.105 ± 0.024 and for PTV 1.335 ± 0.016) respectively, with statistically significance (*p *< 0.01).

**Table 3:** Comparisons of CI and HI for Pgtv and PTV in the present study (n = 10).

Patient number	Pgtv				PTV			
	
	CI		HI		CI		HI	
	10 mm	4 mm	10 mm	4 mm	10 mm	4 mm	10 mm	4 mm
**1**	0.709	0.740	1.090	1.083	0.684	0.672	1.357	1.341
**2**	0.665	0.690	1.094	1.087	0.676	0.688	1.347	1.323
**3**	0.721	0.721	1.090	1.086	0.725	0.734	1.318	1.304
**4**	0.689	0.725	1.095	1.074	0.708	0.723	1.337	1.317
**5**	0.438	0.553	1.108	1.087	0.668	0.677	1.351	1.324
**6**	0.684	0.706	1.145	1.134	0.718	0.734	1.330	1.310
**7**	0.720	0.737	1.151	1.128	0.708	0.728	1.349	1.320
**8**	0.699	0.730	1.093	1.087	0.652	0.671	1.311	1.296
**9**	0.719	0.734	1.080	1.074	0.700	0.699	1.328	1.312
**10**	0.722	0.727	1.101	1.089	0.750	0.748	1.322	1.302
**Average (mean ± SD)**	0.677 ± 0.086	0.706 ± 0.056	1.105 ± 0.024	1.093 ± 0.021	0.699 ± 0.029	0.707 ± 0.029	1.335 ± 0.016	1.315 ± 0.013
***p *value**	0.008		0.001		0.03		0.007	

CI: conformity index; HI: homogeneous index; Pgtv: planning gross target volume; PTV: planning target volume; 10 and 4 mm: multileaf collimator leaf widths as 10 and 4 mm, respectively.

### Dose Sparing of the OARs

Table 4 showed the comparisons of the DVH-based parameters of the spinal cord in the present study. Compared with the plans with the sMLC, the plans with mMLC had significant advantages in dose sparing of the spinal cord. The differences of the D_5 _and gEUD between the two series were statistical significant, respectively (*p *< 0.05). However, there was no statistical significance observed among the differences of the Dmax of the spinal cord between the two series (*p *> 0.05).

**Table 4:** Comparisons of the DVH-based parameters of OARs in the present study (n = 10).

DVH-based parameters	MLC leaf width (mm)	mean ± SD	*p *value
***Spinal cord***			
Dmax (cGy)	10	4288.5 ± 333.9	0.20
	4	4159.1 ± 422.5	
D_5 _(cGy)	10	3404.5 ± 374.4	0.021
	4	3260.3 ± 374.0	
gEUD (cGy)	10	1849.2 ± 297.6	0.049
	4	1815.1 ± 281.7	
***Total lungs***			
V_5 _(%)	10	43.2 ± 6.9	0.48
	4	42.9 ± 6.6	
V_10 _(%)	10	34.0 ± 6.7	0.004
	4	33.2 ± 6.5	
V_20 _(%)	10	16.6 ± 4.7	0.004
	4	16.0 ± 4.6	
V_30 _(%)	10	5.8 ± 2.3	0.09
	4	5.4 ± 2.0	
MLD (cGy)	10	887.9 ± 172.1	0.005
	4	866.2 ± 174.1	
gEUD (cGy)	10	956.8 ± 171.0	0.017
	938.6 ± 175.2		

DVH: dose volume histogram; OARs: organs at risk; MLC: multileaf collimator; Dmean and Dmax: average and maximum irradiation dose; D_5_: irradiation dose received by the 5% of volume of OAR; gEUD: generalized equivalent uniform dose; V_5_,_10_,_20_,_30_: the volume of total lungs receiving the dose of 5, 10, 20 and 30 Gy respectively; MLD: mean lung dose.

Meanwhile, table 4 also showed the comparisons of the dosimetric parameters of the lungs in detail, in the present study. Although the differences of the V_5 _and V_30 _between the two series were not statistically significant, the IMRT plans with the mMLC had clearly advantages in the differences of the V_10 _(33.2 ± 6.5 *vs *34.0 ± 6.7, *p *< 0.01), V_20 _(16.0 ± 4.6 *vs *16.6 ± 4.7, *p *< 0.01), MLD (866.2 ± 174.1 *vs *887.9 ± 172.1, *p *< 0.01) and gEUD (938.6 ± 175.2 *vs *956.8 ± 171.0, *p *< 0.02) respectively, compared with those with the sMLC.

## Discussions

For the first time, we initiated a study focusing on the dosimetric differences between the two kinds of IMRT plans for UTEC with the 10-mm leaf width MLC and 4-mm leaf width MLC The results of this study indicated that the small leaf widths could improve conformity of the targets and OARs.

It is intuitive that a smaller leaf width MLC should result in a better beam shaping. To achieve this goal, the smaller leaf width MLC, commonly called "micro-" or "mini"-MLC (mMLC) and with the leaf widths between 1.6 and 4.5 mm defined at the isocenter, were designed and developed [16-20]. Several studies on the dosimetric impacts of MLC leaf widths were conducted and have confirmed dosimetric advantage in conformal radiotherapy and IMRT planning for different tumors with smaller leaves. Kubo *et al. *firstly compared the conformity of 3 D conformal planning using a 1.7 mm leaf width, a 3 mm leaf-width, and 10 mm leaf-width MLCs [21]. Monk *et al. *evaluated the 3-mm and 5-mm MLC for intracranial radiosurgery and found that the 3-mm leaf width MLC improves targets' conformity, although the quantitative differences may not be clinically significant for some cases [6]. Dvorak *et al. *reported that the micro-MLC showed significantly better conformity values compared with the standard IMRT plans using a regular MLC, they compared 10 mm and 3 mm leaf width MLC in stereotactic body radiotherapy of liver and lung lesions [22]. Jin and colleagues reported that the 3-mm MLC had a better dose conformity in treatment plans than those of the 5 and 10-mm MLCs [8]. To be similar with these studies, our data demonstrated that in IMRT treatment for UTEC, the plans with mMLC had the optimal dose coverage for targets (CI: Pgtv 0.706 ± 0.056 and PTV 0.707 ± 0.029) and better dose homogeneity (HI: Pgtv 1.093 ± 0.021 and PTV 1.315 ± 0.013), than the plans with the sMLC (CI: Pgtv 0.677 ± 0.086 and PTV 0.699 ± 0.029; HI: Pgtv 1.105 ± 0.024 and PTV 1.335 ± 0.016) with statistically significance (*p *< 0.05). As mentioned above, data from the previous and present studies stated that the smaller MLC leaf widths are, the better the dose optimization of an IMRT plan would be. However, on the basis of these literatures, it is definite that the impact of the MLC leaf width on IMRT planning and delivery depends on the size and shape of the targets.

There have been several studies that have evaluated the mMLC to improve the surrounding normal tissue sparing, compared with the sMLC. Wang *et al. *observed that there would be the significant reductions in the volume of rectum receiving medium to higher doses in IMRT plans with the mMLC for prostate cancer. In IMRT plans with mMLC, the average decrease in the volume of the rectum receiving 40, 50, and 60 Gy was 40.2%, 33.4%, and 17.7%, respectively. The mean dose reductions for D_17 _and D_35 _for the rectum were 20.0% and 18.3%, respectively, compared with the plans with the sMLC [9]. Wu *et al. *found that in radiosurgery for the small lesions, the complex target-OARs space structures would especially benefit from the use of a smaller leaf-width MLC [7]. The same conclusions were reported by other researchers [8,11,12,23]. In our study, we observed that the plans with the mMLC had apparent advantage on dose sparing of the spinal cord (D_5 _and gEUD), especially when target was very close to the spinal cord, compared with the plans with the sMLC,. For lungs, the mMLC was still superior to the sMLC in the dose sparing in the V_10_, V_20_, MLD and gEUD. Although these differences we observed were somewhat small or even ignored in practice, our results still revealed that the mMLC has more advantages than the sMLC and represented the ongoing progress of the precise radiotherapy techniques.

The limitation of this study should also be addressed. In plan generation, the DMPO method was applied. During the first optimization step for 2-step optimization, the optimizer generates a continuous intensity-modulated profile for each user-selected beam while minimizing the value of the cost function with in a set number of total iterations [23]. The IMRT optimization in Pinnacle with DMPO starts with a conformal beam of uniform intensity followed by four steps of fluence optimization. This is followed by a step that includes machine parameters: leaf-positions and segment weights are varied within the limits of the linear accelerator. With DMPO, there are additional parameters that can be defined by the treatment planner, including the max iterations of plan optimizing, the maximum numbers of all segments and the minimum monitor units (MUs) per segment. Different planner may set up different parameters based on different clinical conditions. Ludlum *et al. *showed that the iterations number is 15 are sufficient for convergence of the cost function for most clinical cases [24]. Asselen *et al. *considered that the maximum number of segments is a soft constraint of the optimization process. In their experiences, the number of initial iterations was 10 [25]. In addition, Ludlum *et al. *and Bratengeiera *et al. *respectively expressed increasing iteration number does not improve the plan quality significantly [24,26]. As our focus in this study was to compare two kinds of MLCs, we directly set up the max iterations of plan optimizing and the maximum numbers of all segments in DMPO optimization based on our daily clinical practice and did not compare the plans with different DMPO steps.

This work has been purely a theoretical planning study and no attempt has been made to measure the dose distribution delivered in practice based on the two MLC devices. The actual physical dose delivered to patients could be affected by many other factors such as beam penumbra modeling, setup uncertainty, organ motions. The clinical significance on these results would deserve further investigation. It is noteworthy that the MLC leaf width is not the only parameter affecting the IMRT plans. Other factors could also be included, such as the leaf transmission, leakage radiation, the source MLC distance.

## Conclusions

The mMLC not only resulted in a dosimetric improvement in OARs' protection in the IMRT plans for UTEC compared with the sMLC, but also ensured the improvement of the dose delivery efficiency and the target dose coverage. Because of its superior ability to spare normal tissues, the use of sMLC may improve the therapeutic ratio by reducing the toxicity to the adjacent OARs during IMRT delivery.

## Competing interests

The authors declare that they have no competing interests.

## Authors' contributions

YG and SW contributed equally in design of the study, collection of data and drafting the manuscript; LZ, YL, YX, YL, SB, YF, QX and QJ worked on collection of data and critical revision of the manuscript; YG and SW provided the conception of this study and the final approval of the version to be published. And all authors read and approved the final manuscript.
